# Beyond the Amyloid Hypothesis: Systemic Drivers, CNS-PNS Crosstalk, and the Future of Alzheimer’s Disease Therapeutics

**DOI:** 10.3390/ijms27115042

**Published:** 2026-06-02

**Authors:** Amador Velázquez de Castro-Bono, Gracia Castro-Luna, José Luis Guil-Guerrero

**Affiliations:** 1 Hospital Central de la Defensa “Gómez Ulla”, 28047 Madrid, Spain; avelazquezcb@gmail.com; 2 Department of Nursing, Physiotherapy and Medicine, University of Almería, 04120 Almería, Spain; graciacl@ual.es; 3 Food Technology Division, University of Almería, 04120 Almería, Spain

**Keywords:** Alzheimer’s disease, cognitive decline, neuroinflammation, microbiota–gut–brain axis, systemic neurodegeneration, brain insulin resistance, disease-modifying therapies

## Abstract

Alzheimer’s disease (AD) is undergoing a profound paradigm shift, transitioning from a localized, monolithic proteinopathy into a complex, multisystem disorder. This critical review synthesizes recent mechanistic, translational, and clinical insights to dismantle the traditional linear amyloid cascade hypothesis. We explore the synergistic interplay between amyloid-β (Aβ) and tau propagation, positioning chronic neuroinflammation, endolysosomal failure, and metabolic starvation—often framed as “Type 3 Diabetes”—as fundamental disease drivers. Crucially, we highlight the emerging biological bridge of CNS-PNS crosstalk, where central neurodegeneration and peripheral neuropathies are linked by systemic immune activation and microbiota–gut–brain axis dysbiosis. The recent validation of disease-modifying therapies (DMTs) confirms Aβ clearance as a viable pharmacological target; however, the marginal clinical gains and severe radiological risks, such as Amyloid-Related Imaging Abnormalities (ARIA), expose the profound limitations of monotherapy. Ultimately, we argue that isolated amyloid clearance is merely an induction phase. The future of AD therapeutics mandates a sequential combination approach—pairing early plaque debulking with lifelong metabolic and neuroimmune maintenance. Supported by scalable fluid biomarkers (e.g., plasma p-tau217) and the expanded ATN(I) framework, the field must embrace proactive precision medicine and inclusive clinical trial designs to successfully transform AD into a manageable chronic condition.

## 1. Introduction: The Biological Reconceptualisation of Alzheimer’s Disease

For over a century, Alzheimer’s disease (AD) was restrictively defined by its end-stage clinical manifestations and the post-mortem observation of its pathological hallmarks: extracellular amyloid-beta (Aβ) plaques and intracellular neurofibrillary tau tangles [1]. Mechanistically, these hallmarks originate from the aberrant cleavage of the transmembrane amyloid precursor protein (APP). While physiological processing of APP follows a benign, non-amyloidogenic pathway, pathological shifts in secretase enzyme activity generate aggregation-prone Aβ peptides. These neurotoxic by-products accumulate to form extracellular plaques, which in turn trigger cascading downstream events—most notably the hyperphosphorylation of tau proteins that ultimately collapse into intracellular neurofibrillary tangles. This clinical-pathological view heavily entrenched the “amyloid cascade hypothesis” as the singular dogma of neurodegeneration, driving decades of clinical trials that largely failed [2]. These historic setbacks were not necessarily due to an incorrect target, but rather a profound misunderstanding of disease staging and a failure to recognize AD as a highly complex, systemic disorder.

Today, the therapeutic landscape has undergone a definitive paradigm shift. The conceptualisation of AD has transitioned from a subjective, symptom-based syndrome to a measurable, biological construct. Driven by the National Institute on Aging–Alzheimer’s Association (NIA-AA) AT(N) framework—and its more recent evolution into the ATN(I) model [3]—the field now relies on advanced neuroimaging (PET/MRI) and highly sensitive fluid biomarkers to detect Amyloid (A), Tau (T), Neurodegeneration (N), and Neuroinflammation (I) in vivo, often decades before clinical symptoms arise [4,5].

This biomarker revolution has directly facilitated the recent regulatory approvals of disease-modifying therapies (DMTs), specifically anti-amyloid monoclonal antibodies such as lecanemab and donanemab [6]. These agents must be accurately contextualized within the broader treatment landscape: although they do not represent a definitive cure for AD, they currently constitute promising therapeutic strategies capable of slowing cognitive decline in carefully selected patients. By validating amyloid clearance as a highly predictive surrogate endpoint, these agents represent a historic milestone. However, they concurrently expose the severe limitations of targeting a single protein. The modest clinical gains of these therapies, paired with significant radiological safety burdens—namely Amyloid-Related Imaging Abnormalities (ARIA)—demonstrate that while amyloid may be the initial trigger, it does not act in isolation [7].

Increasingly, the myopic focus on isolated cerebral proteinopathy is giving way to a broader understanding of AD as a systemic failure of cellular communication and bioenergetic regulation. To truly alter the disease course, therapeutic models must address the dense crosstalk between the central and peripheral nervous systems (the CNS-PNS axis). The AD brain is actively besieged by innate neuroinflammation, profound endolysosomal dysfunction, and localized insulin resistance—a metabolic starvation profile so severe it was famously proposed as “Type 3 Diabetes” by de la Monte and colleagues [8].

This review critically evaluates the modern AD framework. We analyze the indispensable role of the neuroradiologist in navigating the efficacy and safety of new DMTs, explore the systemic drivers of peripheral and central neurodegeneration, and outline the urgent transition toward multi-omic profiling. Ultimately, this manuscript argues that the future of Alzheimer’s therapeutics lies not in isolated protein clearance, but in multimodal, precision-medicine combination therapies designed to transform a terminal cognitive decline into a manageable chronic condition.

## 2. Prevalence and Epidemiology

### 2.1. Prevalence, Comorbid Patterns, and Impact on Cognition, Function, and Quality of Life

AD is a progressive neurodegenerative disorder and the predominant etiology of dementia worldwide [9]. Currently, over 55 million individuals globally are living with dementia, a prevalence projected to surpass 152 million by 2050 as a direct consequence of an aging global demographic [10]. In the United States alone, the disease affects nearly 7 million adults aged 65 and older, constituting a critical public health crisis associated with a profound socioeconomic burden [11].

Crucially, “pure” AD pathology is increasingly recognized as the clinical exception rather than the rule. The condition frequently presents as “mixed dementia,” characterized by a complex interplay with cerebrovascular disease [12]. Prevalent systemic comorbidities—most notably hypertension, type 2 diabetes mellitus, and broad cardiovascular disease—significantly exacerbate neurovascular unit dysfunction and accelerate the trajectory of cognitive decline [13]. Furthermore, neuropsychiatric comorbidities, including depression, apathy, and anxiety, frequently manifest during the prodromal and early clinical stages, functioning dually as core phenotypic features and catalysts for further deterioration [14]. However, a profound discrepancy exists between this epidemiological reality and contemporary clinical trial design. While population data confirm that AD overwhelmingly co-occurs with vascular and metabolic comorbidities, pivotal disease-modifying therapy (DMT) trials systematically exclude these exact patients to isolate pure amyloid pathology [15]. Consequently, a massive translational knowledge gap remains regarding the safety and efficacy of emerging biologics in the highly comorbid, real-world aging population.

The clinical progression of AD entails a relentless deterioration across multiple cognitive domains. This typically initiates with episodic memory deficits before advancing to profoundly impair executive function, visuospatial processing, and language capabilities [16]. Such cognitive erosion directly translates into severe functional disability, systematically compromising the patient’s capacity to execute both basic Activities of Daily Living (ADLs)—such as dressing and feeding—and more complex Instrumental Activities of Daily Living (IADLs), such as managing finances, medication, or transportation. To accurately evaluate and monitor this trajectory of cognitive impairment and neurodegeneration, clinical practice relies heavily on widely used standardized clinical assessment tools. Specifically, the Mini-Mental State Examination (MMSE) serves as a foundational instrument for quantifying global cognitive decline, while validated ADL and IADL scales are explicitly utilized to stage the severity of real-world functional loss. This progressive loss of autonomy, invariably compounded by behavioral and psychological symptoms of dementia (BPSD), imposes a devastating emotional, physical, and financial toll on caregivers, underscoring the urgent imperative for holistic care models [17].

### 2.2. Key Patient Subgroups and Risk Modifiers

The epidemiological landscape of AD is notably heterogeneous. Although advancing age remains the primary risk factor, sex and race function as critical disease stratifiers [11]. Women account for approximately two-thirds of all AD cases; this prevalence disparity is only partially attributable to greater female longevity, pointing toward unique biological, metabolic, and neuroendocrinological vulnerabilities [11]. As highlighted by Andrew and Tierney [18], the etiology of this sex-based disparity is highly complex and cannot be reduced to a simple survival artifact. Instead, it is driven by an intersection of biological vulnerabilities and sociocultural determinants, including the profound metabolic shifts associated with the menopausal transition—which abruptly deprives the central nervous system of estrogen’s neuroprotective and bioenergetic support. Additionally, women historically exhibit differences in the accumulation of cognitive reserve and demonstrate higher rates of multidimensional frailty at equivalent ages compared to men, all of which lower the clinical threshold for dementia manifestation [18].

Furthermore, racial and ethnic minorities exhibit markedly divergent incidence rates. Specifically, older Black Americans are approximately twice as likely, and older Hispanic Americans are about one and a half times as likely, to develop AD compared to older non-Hispanic White populations [11]. These disparities are driven by a confluence of compounding factors, including higher burdens of cardiovascular comorbidities and structurally differential access to care [11]**.** Despite bearing this substantially higher relative risk, Black and Hispanic populations remain chronically underrepresented in neuroimaging, fluid biomarker, and genomic research, frequently comprising less than 5% of pivotal Phase 3 trial cohorts [19,20]. This creates a dangerous void in our understanding of how the ATN(I) biomarker framework, Apolipoprotein E gene (APOE) ε4 penetrance, and DMT safety profiles (such as ARIA risk) manifest across diverse genetic and socioeconomic backgrounds.

The etiology of AD is further modulated by a complex interplay of genetic predispositions and environmental exposures. While the APOE ε4 allele remains the most potent genetic risk modifier, an estimated 40% of dementia cases worldwide are attributable to modifiable risk factors [21]. The Lancet Commission has delineated a life-course model detailing these targets, encompassing early-life educational attainment, mid-life hearing loss, hypertension, and obesity, alongside late-life factors such as depression, physical inactivity, diabetes mellitus, and social isolation [21]. Nevertheless, a distinct mechanistic disconnect persists in current research. While epidemiological models attribute a massive 40% of dementia risk to these modifiable factors [21], there is a glaring lack of longitudinal data directly linking these systemic exposures to the molecular acceleration of amyloid, tau, and neuroinflammation. Bridging the gap between macro-level public health interventions and micro-level biomarker validation remains a critical priority for future preventative trial designs [22].

## 3. Mechanistic Insights of Alzheimer

### 3.1. Proteinopathies: Amyloid-β, Tau, and Synaptic Dysfunction

Alzheimer’s disease is classically defined by the accumulation of amyloid-β (Aβ) plaques and tau neurofibrillary tangles; however, conceptualizing these proteinopathies merely as parallel events is an oversimplification. Rather, Aβ and tau drive a synergistic, multi-cellular cascade ultimately culminating in profound synaptic failure—a disruption of neurotransmission and synaptic plasticity that serves as the most direct structural correlate of cognitive decline [23]. Furthermore, the progressive deterioration of these neural networks underscores the necessity of moving beyond amyloid-centric models to address the broader bioenergetic and synaptic collapse defining the disease state.

The pathogenic cascade is largely initiated by the aberrant processing of the amyloid precursor protein (APP), generating soluble Aβ oligomers. Crucially, it is these highly mobile oligomers, rather than insoluble plaques, that are now recognized as the primary neurotoxic species. They directly disrupt synaptic signaling and plasticity, precipitating early synaptic failure well before overt neuronal apoptosis occurs [24]. Beyond direct synaptotoxicity, Aβ oligomers act as potent inflammatory stimuli, triggering pathological microglial and astrocytic activation. This establishes a maladaptive neuroinflammatory milieu that not only exacerbates neuronal injury but also severely compromises intrinsic Aβ clearance mechanisms, locking the brain into a self-perpetuating cycle of toxicity [25].

A fundamental pillar of this clearance infrastructure is the glymphatic system, a brain-wide paravascular pathway for waste clearance. This system is facilitated by Aquaporin-4 (AQP4) water channels located on astrocytic endfeet, which allow the exchange of cerebrospinal fluid (CSF) and interstitial fluid (ISF) to flush out neurotoxic proteins such as Aβ and tau. When this clearance interface is compromised, Aβ serves as a critical upstream trigger for tau pathology, which is widely considered the primary executioner of neurodegeneration [26].

In the AD milieu, a critical imbalance between kinase and phosphatase activity drives aberrant tau hyperphosphorylation. This causes tau to dissociate from microtubules and self-assemble into toxic intracellular tangles, effectively dismantling axonal transport corridors. Notably, the spatiotemporal progression of tau pathology—unlike Aβ plaque burden—exhibits a robust and direct correlation with the severity of cognitive impairment [27]. Synaptic dysfunction serves as the critical convergence point where Aβ and tau pathologies synergistically dismantle neural networks. Compelling evidence demonstrates that tau acts as an essential downstream mediator of Aβ-induced synaptotoxicity; without dendritic tau, the deleterious effects of Aβ oligomers are significantly blunted [28].

### 3.2. Neuroinflammation and CNS–PNS Crosstalk

Emerging evidence supports a profound, bidirectional pathophysiological relationship between AD and peripheral neuropathies. Historically compartmentalized as anatomically distinct disorders, these conditions are now recognized as interconnected manifestations of systemic neuroinflammation.

#### 3.2.1. The Core Mechanism: Neuroinflammation

Chronic neuroinflammation serves as the principal bridge linking AD and peripheral neuropathies. Within the CNS, microglia orchestrate the innate immune response, whereas resident macrophages and Schwann cells fulfill analogous roles in the PNS [29]. In neurodegeneration, these populations undergo a parallel phenotypic shift to neurotoxic, pro-inflammatory profiles, driven by a shared milieu of cytokines (Tumor necrosis factor-α (TNF-α) and Interleukin (IL) 1β and Interleukin 6 (IL-1β and IL-6)) [30]. When elevated in the periphery, these cytokines compromise the blood–brain barrier (BBB), permitting peripheral inflammatory mediators to infiltrate the CNS [31]. However, a critical spatiotemporal disconnect persists in current longitudinal models. While it is evident that systemic metabolic stress and peripheral inflammation precede overt central pathology, the exact temporal inflection point—the moment peripheral meta-inflammation permanently “flips the switch” to drive irreversible central microglial priming—remains unmapped [32]. Clarifying this chronological sequence is vital for determining the optimal window for early immune-modulating interventions.

#### 3.2.2. CNS–PNS Crosstalk Pathways

**Figure 1:**
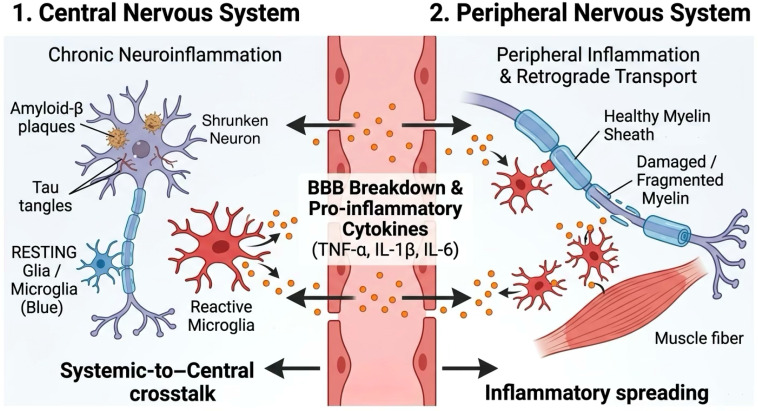
The Neuroinflammatory Loop and Systemic Crosstalk Between CNS and PNS. This diagram illustrates the bidirectional exacerbation of central and peripheral neurodegeneration, driven by systemic cytokines and immune factors traversing a compromised blood–brain barrier.

A primary conduit for neuro-immune communication is the vagus nerve. In AD, progressive attenuation of vagal tone impairs endogenous anti-inflammatory regulation, permitting peripheral inflammation to propagate unchecked [33]. Furthermore, neuronal survival hinges upon microtubule-dependent transport, which fails profoundly in both AD and peripheral neuropathies. The failure of molecular motors (dynein and kinesin) to deliver Nerve Growth Factor (NGF) precipitates somatic starvation and a characteristic “dying-back” axonopathy. This dynamic, bidirectional neuroinflammatory loop is illustrated in Figure 1.

#### 3.2.3. Clinical Intersections and Biomarkers

This neuro-immune crosstalk clarifies why peripheral motor deficits—such as altered gait biomechanics—often predate amnesic symptoms. Fluid biomarkers such as Neurofilament Light Chain (NfL) and Brain-Derived Neurotrophic Factor (BDNF) collectively reflect this pan-axonal degradation. In neurodegenerative conditions, NfL levels are markedly increased in both CSF and blood, reflecting the structural breakdown of the axonal cytoskeleton and the subsequent release of neurofilament proteins into the extracellular space [34]. Conversely, BDNF levels are typically reduced, indicating impaired neurotrophic support and diminished neuronal resilience [35]. This reduction in BDNF is mechanistically driven by chronic neuroinflammation, which downregulates BDNF transcription and impedes its axonal transport, leaving neurons highly vulnerable to toxic insults [36]. Furthermore, BDNF plays a critical, foundational role in the mechanisms of learning and working memory by modulating synaptic plasticity and sustaining long-term potentiation (LTP). Beyond its core cognitive functions, disruptions in BDNF signaling and expression have been strongly implicated in the pathophysiology of several psychiatric disorders, including major depressive disorders and schizophrenia. This highlights BDNF’s broad neurotrophic influence across both cognitive and affective domains, inextricably linking mood dysregulation with cognitive decline in neurodegenerative trajectories [37].

### 3.3. Alzheimer’s Disease and Neuropathies: Mitochondrial and Axonal Vulnerability

Mitochondrial dysfunction is a primary, initiating driver of systemic neuronal vulnerability. Impairments in bioenergetics, compromised calcium homeostasis, and elevated reactive oxygen species (ROS) act synergistically to precipitate early synaptic failure [38]. Because axons possess massive metabolic demands yet limited regenerative capacities, accumulating Aβ oligomers and hyperphosphorylated tau catastrophically disrupt the bidirectional transport of mitochondria [39]. Consequently, mitochondria fail to reach distal synaptic terminals.

Critically, because peripheral neurons possess exceptionally long axons and lie outside the protective blood–brain barrier (BBB) [40], they are highly susceptible to the systemic inflammatory mediators and metabolic stressors that drive this transport failure [41]. This anatomical vulnerability suggests that peripheral nerve damage is not merely a parallel comorbidity, but can serve as an early, clinically accessible indicator of central neurodegenerative processes. The local energy crisis that drives the retrograde “dying-back” axonopathy observed in peripheral neuropathies perfectly mirrors—and may clinically precede—the large-scale network disconnection characteristic of advanced AD [42]. Therefore, the manifestation of peripheral neuropathy provides a vital diagnostic window into the systemic mitochondrial collapse and neuroinflammation that are simultaneously threatening the central nervous system.

### 3.4. Vascular/Metabolic Stress and Systemic Modifiers

#### 3.4.1. Vascular and Metabolic Stress: The Critical Role of Circulation and the BBB

The cardiovascular system’s role in neurodegeneration extends far beyond acting as a mere conduit in the gut–brain axis; it is a fundamental prerequisite for maintaining central homeostasis. The brain possesses massive energy demands and relies entirely on continuous macro- and microvascular perfusion. Consequently, cerebrovascular pathology—ranging from large-vessel atherosclerosis to microvascular endothelial dysfunction—is increasingly recognized not just as a comorbidity, but as an early, predictive indicator and driver of most known neurodegenerative diseases, including Alzheimer’s disease (AD) [43].

A cornerstone of this circulatory impairment is the disruption of the blood–brain barrier (BBB). In a healthy state, the tightly regulated neurovascular unit and the BBB work synergistically to upkeep brain homeostasis, facilitating nutrient transport while shielding the brain from neurotoxic blood-derived pathogens and systemic inflammation. However, poor cardiovascular health and aging compromise this structural integrity, increasing BBB permeability [44]. To capture this early vascular pathology, emerging clinical biomarkers such as soluble platelet-derived growth factor receptor beta (sPDGFRβ)—a specific indicator of pericyte injury and BBB breakdown—are becoming essential tools for precision diagnostics [45]. Monitoring such markers allows for the detection of neurovascular deterioration well before the onset of irreversible cognitive decline**.** This vascular leakiness allows the uncontrolled influx of neurotoxic plasma proteins, immune cells, and inflammatory cytokines into the central nervous system. This breach creates a persistent neuroinflammatory environment that is now understood to precede and accelerate Aβ and tau accumulation [46].

This systemic vascular collapse acts synergistically with metabolic dysregulation. The conceptualisation of AD as “Type 3 Diabetes” describes a brain-specific manifestation of insulin resistance that fundamentally impairs neuronal bioenergetics [47]. The desensitization of central insulin receptors precipitates an acute state of neuronal “energy starvation,” directly disinhibiting downstream kinases and exacerbating tau hyperphosphorylation [48]. Concurrently, this metabolic failure exacerbates microvascular deterioration, further compromising BBB integrity and trapping the brain in a vicious, self-amplifying cycle of vascular hypoperfusion, energy starvation, and protein aggregation.

#### 3.4.2. Systemic Modifiers: The Microbiota–Gut–Brain Axis (MGBA)

Beyond the cranium, the bidirectional Microbiota–Gut–Brain Axis (MGBA) functions as a critical third pillar of neuro-immune regulation. A diet- or pathology-induced microbial imbalance—characterized by a reduction in beneficial, short-chain fatty acid (SCFA)-producing microbiota and an overgrowth of Gram-negative opportunistic bacteria—drives intestinal hyperpermeability (“leaky gut”) [49,50]. The downregulation of tight junction proteins (e.g., claudin, occludin) allows the systemic translocation of neurotoxic microbial products, such as lipopolysaccharides (LPS) and pro-inflammatory cytokines, into the bloodstream [51].

Once systemic, this peripheral inflammatory burden interacts with bidirectional vagal communication, where afferent gut-derived signaling and efferent anti-inflammatory responses attempt to mediate the gut–brain interface. Furthermore, the gut microbiota synthesizes essential neurotransmitters (e.g., serotonin, GABA) and hormones that directly influence central neurochemistry [52,53]. The profound impact of this system is highlighted by the emerging field of “psychobiotics”—specific live bacterial strains that have provided robust clinical evidence for alleviating anxiety and depression, thereby firmly establishing the MGBA’s role in psychiatric and neurodegenerative trajectories [54,55].

**Figure 2:**
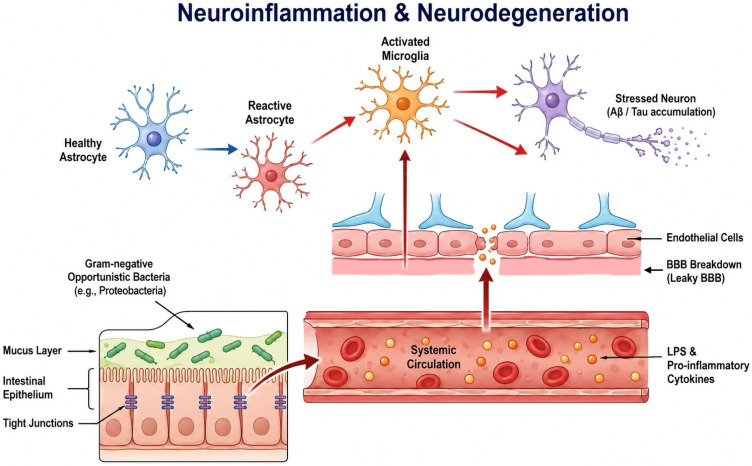
The Comprehensive Microbiota–Gut–Brain Axis (MGBA). Systemic Pathways to Neuroinflammation and Neurodegeneration. This schematic illustrates the cascade linking gut dysbiosis and intestinal hyperpermeability (“leaky gut”) to blood–brain barrier (BBB) breakdown and central neurodegeneration. Intestinal dysbiosis drives the overproduction of endotoxins (e.g., LPS) while depleting neuroprotective metabolites. As intestinal tight junctions degrade, these microbial toxins enter the bloodstream, triggering chronic systemic inflammation and aberrant vagal nerve signaling. This peripheral inflammatory burden ultimately compromises BBB integrity, allowing neurotoxins to infiltrate the brain parenchyma. Consequently, this drives chronic microglial activation and astrogliosis, synergistically accelerating amyloid-beta (Aβ) deposition, tau hyperphosphorylation, and progressive synaptic loss.

Ultimately, circulating systemic mediators compromise BBB integrity via increased matrix metalloproteinases (MMPs) and astrocyte end-foot retraction, infiltrating the CNS to force resting M0 microglia into aggressively neurotoxic M1 states (Figure 2). Despite this overwhelming mechanistic evidence that AD is a systemic, multi-organ disorder [56,57], our premier diagnostic criteria—the ATN(I) framework—remains almost exclusively neurocentric. There is a glaring absence of validated peripheral biomarkers (such as gut permeability markers or peripheral cytokine panels) integrated into formal AD staging [58].

#### 3.4.3. Systemic Modifiers: Inflammaging and Peripheral-Central Immune Crosstalk

While the MGBA represents a distinct microbial-host interface, it operates concurrently with the compounding backdrop of “inflammaging”—the chronic, low-grade, sterile systemic inflammation characteristic of advancing age [59]. Unlike gut dysbiosis, inflammaging is primarily driven by immunosenescence and the systemic accumulation of senescent cells that secrete a toxic cocktail of pro-inflammatory cytokines, known as the senescence-associated secretory phenotype (SASP) [60].

When the age-related burden of inflammaging converges with gut-derived LPS, the resulting systemic inflammatory storm profoundly compromises BBB integrity. Circulating systemic mediators degrade the barrier via increased matrix metalloproteinases (MMPs) and the induction of astrocyte end-foot retraction [61]. This vascular leakiness allows peripheral immune cells and cytokines to infiltrate the CNS, forcing resting homeostatic microglia into aggressively neurotoxic, pro-inflammatory states (Figure 2).

### 3.5. Genetic Contributions and Gene–Environment Interactions

The genetic architecture of AD is fundamentally divided into early-onset (EOAD) and late-onset (LOAD) forms, each dictated by distinct pathogenic trajectories [62]. This architecture is deeply complex, necessitating a nuanced understanding of genotype–phenotype correlations, zygosity effects, penetrance, and expressive variability**.** EOAD, which accounts for a small minority of cases (typically presenting before age 65), is primarily driven by highly penetrant, autosomal dominant mutations in the amyloid precursor protein (*APP*), *presenilin-1* (*PSEN1*), and *presenilin-2* (*PSEN2*) genes. In these familial cases, the genetic defect alone is usually sufficient to aggressively drive early amyloidosis and disease onset. However, clinical penetrance and phenotypic expressivity can vary significantly even among individuals harboring the same mutation [63].

To provide a rigorous, multi-scale overview of these distinct genetic architectures, Table 1 details the primary genetic determinants implicated in both EOAD and LOAD. This comprehensive compilation explicitly charts their chromosomal localizations, official Online Mendelian Inheritance in Man (OMIM) associations, core disease classifications, and the profound phenotypic variability observed in clinical practice.

Furthermore, the genetic boundaries between distinct neurodegenerative syndromes are increasingly recognized as fluid. For instance, while hexanucleotide repeat expansions in the *C9orf72* gene are classically associated with frontotemporal dementia (FTD) and amyotrophic lateral sclerosis (ALS), recent literature has increasingly reported their association with AD-like clinical phenotypes, highlighting the pleiotropic nature of dementia genetics [64].

To unravel this intricate genetic landscape and identify individuals at risk of early-onset cognitive impairment, modern Next-Generation Sequencing (NGS) diagnostic approaches have become indispensable in clinical and research settings. Techniques such as Whole Exome Sequencing (WES), Whole Genome Sequencing (WGS), and targeted gene panels provide high-resolution mapping of both rare pathogenic variants and complex polygenic risk profiles. These advanced genomic tools are crucial for comprehensively understanding the genetic architecture of AD, enabling early diagnostic stratification and facilitating precision-medicine therapeutic trials [65,66].

While autosomal dominant mutations dictate the aggressive onset of familial disease, the polygenic risk factors underlying sporadic LOAD function quite differently—they do not act as independent determiners but rather establish the baseline physiological vulnerability required for subsequent gene–environment interactions. For example, the *APOE ε4* allele remains the most potent genetic risk modifier for LOAD, where zygosity exerts a profound effect: homozygous *APOE ε4* carriers exhibit a significantly accelerated age of onset and a more aggressive clinical phenotype compared to heterozygotes [67].

**Table 1:** Genetic determinants of early- and late-onset Alzheimer’s disease.

Gene	Chromosome Location	OMIM ID	Disease Classification	Clinical Significance and Phenotypic Variability	References
*APP* (*Amyloid Precursor Protein*)	21q21.3	#104760	Early-Onset AD (Autosomal Dominant)	Alters proteolytic cleavage to increase the ratio of neurotoxic Aβ42/Aβ40. Phenotype exhibits complete penetrance with symptom onset typically between ages 45 and 65.	[68,69]
*PSEN1* (*Presenilin 1*)	14q24.2	#104311	Early-Onset AD (Autosomal Dominant)	Accounts for up to 70% of familial EOAD cases. Mutations cause loss-of-function in the γ-secretase catalytic subunit, shifting cleavage toward longer, more aggregable Aβ peptides. Highly aggressive; onset can be as early as the 30 s.	[70,71]
*PSEN2* (*Presenilin 2*)	1q42.13	#600759	Early-Onset AD (Autosomal Dominant)	Rare structural component/modifier of the γ-secretase complex. Exhibits highly variable, incomplete penetrance and a wider range of symptom onset (ages 40–85) compared to PSEN1.	[72,73]
*APOE* (*Apolipoprotein E*)	19q13.32	#107741	Late-Onset AD (Susceptibility Risk Factor)	The ε4 allele is the strongest genetic risk factor for sporadic LOAD. It severely impairs peripheral/central lipid recycling, disrupts blood–brain barrier (BBB) pericyte integrity, and hinders central amyloid clearance.	[67,74]
*TREM2* (*Triggering Receptor Expressed on Myeloid Cells 2*)	6p21.1	#605086	Late-Onset AD (Susceptibility Risk Factor)	Rare heterozygous missense variants (e.g., R47H) significantly increase LOAD risk by impairing microglial survival, proliferation, and metabolic transition into a reparative state around plaques.	[75,76]
*ABCA1* (*ATP-Binding Cassette Subfamily A Member 1*)	9q31.1	#600046	Late-Onset AD (Risk Modifier)	Regulates cellular cholesterol efflux and the baseline lipidation of APOE. Genetic loss-of-function variants drive poorly lipidated APOE, worsening microvascular damage and accelerating amyloid deposition.	[77,78]
*CLU* (*Clusterin/Apolipoprotein J*)	8p21.1	#185430	Late-Onset AD (Risk Modifier)	Acts as an extracellular chaperone that prevents non-native protein aggregation. Genetic variants alter Aβ clearance kinetics and modulate complement-mediated neuroinflammation across different ethnic cohorts.	[79,80]

# Indicates a confirmed genetic disease phenotype with a known molecular basis in the OMIM database.

In contrast, the vast majority of AD cases are late-onset and polygenic. The etiology of AD is a synergistic convergence of heritable susceptibility and environmental exposures. This convergence occurs when specific genetic vulnerabilities—most notably the *APOE ε4* allele, alongside variants in neuroimmune and lipid-regulating genes such as *Triggering receptor expressed on myeloid cells 2* (*TREM2*), or *ATP-binding cassette subfamily A member 1* (ABCA1)—interact with chronic external stressors (such as obesogenic diets, neurotoxins, or chronic infections) to produce a compounded pathogenic outcome that neither factor could achieve as rapidly on its own [81]. For instance, while the *APOE ε*4 allele severely impairs central amyloid clearance, it simultaneously exacerbates peripheral microvascular degradation by disrupting lipid recycling [82]. When an *APOE ε4* carrier is exposed to an obesogenic diet, these factors synergize to drive severe systemic insulin resistance and systemic cytokine storms (e.g., IL-1β, IL-18).

**Figure 3:**
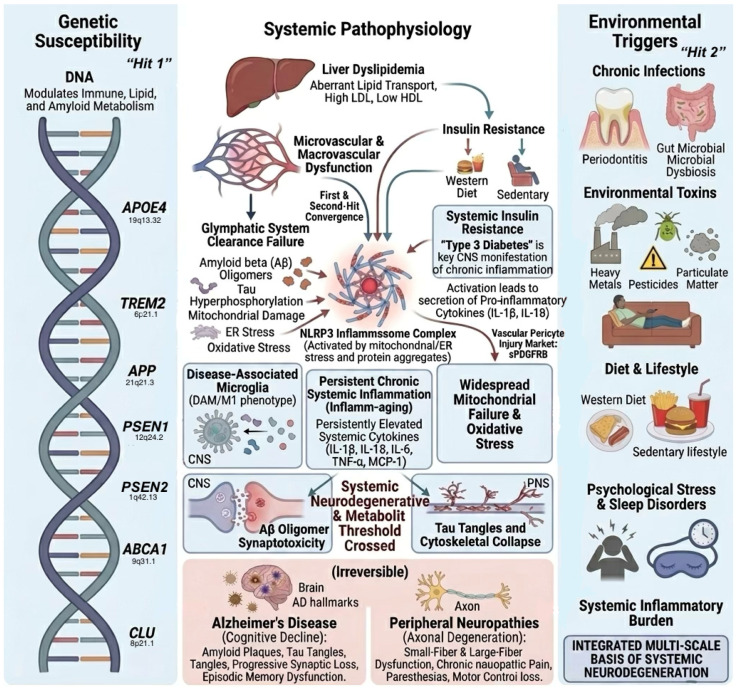
Integrated multi-scale model of systemic neurodegeneration. This diagram illustrates the “double-hit” hypothesis, demonstrating how genetic susceptibility and chronic environmental stressors converge to drive interconnected central and peripheral neurodegeneration. This systemic metabolic and inflammatory collapse is shown as being centrally mediated by a combined hub of systemic inflammation and insulin resistance, which in turn triggers distinct pathogenic cascades in the Central Nervous System (e.g., Microglia/M1 activation, Aβ oligomers, Tau hyperphosphorylation, glymphatic failure) and the Peripheral Nervous System (e.g., macrophage polarization, distal axonal dying-back), culminating in Alzheimer’s Disease and peripheral neuropathies, respectively. Note: *APP*, *PSEN1*, and *PSEN2* mutations classically drive Early-Onset AD (EOAD). Conversely, polygenic risk profiles (*APOE4*, *TREM2*, *ABCA1*, and *CLU*) define Late-Onset AD (LOAD), acting as a primary baseline vulnerability (Hit 1) that requires environmental and systemic stressors (Hit 2) to cross the threshold into full systemic neurodegeneration [41,85].

This dynamic perfectly illustrates the “double-hit” hypothesis: a physiological baseline compromised by genetic susceptibility (the first hit) requires the compounding insult of chronic environmental or metabolic stress (the second hit) to cross the threshold into irreversible neurodegeneration (Figure 3) [83]. This complex gene–environment reality exposes a critical limitation in contemporary research. While single-mutation transgenic murine models (e.g., isolated *APP* mutations) have been undeniably invaluable for isolating specific molecular pathways of amyloid cleavage, they fundamentally fail to replicate the systemic “double-hit” interactions seen in human AD [84]. In human patients, polygenic vulnerability collides with decades of vascular disease, inflammaging, and metabolic syndrome. Consequently, preclinical success in highly controlled models rarely translates to clinical efficacy, highlighting an urgent need for multi-morbidity animal models that accurately reflect the human systemic aging process.

## 4. Biomarkers and Diagnostics

### 4.1. CNS: Plasma and CSF Markers, Neuroimaging (Amyloid/Tau PET, MRI)

The diagnostic paradigm for AD has fundamentally shifted from a symptom-based, clinico-pathological framework to a biologically defined construct. This transition is formalized in the ATN (Amyloid, Tau, and Neurodegeneration) research framework, which utilizes continuous fluid and imaging biomarkers to define the disease state across its preclinical and clinical spectrum, independent of cognitive symptomatology [86]. The rapid integration of ultra-sensitive fluid markers with advanced neuroimaging currently allows for unprecedented precision in early detection, patient stratification, and longitudinal monitoring.

#### 4.1.1. Fluid Biomarkers: CSF and the Plasma Revolution

Cerebrospinal fluid (CSF) analysis has historically served as the “gold standard” for fluid diagnostics, given its direct anatomical communication with the brain’s interstitial space. The classical AD CSF signature is characterized by a reduced amyloid-beta 42/40 (Aβ42/40) ratio—reflecting the cortical sequestration of amyloid into insoluble plaques—coupled with elevated levels of phosphorylated tau (e.g., p-tau181 or p-tau217), which denote active tangle formation [87]. Additionally, non-specific markers of axonal injury, such as Neurofilament Light Chain (NfL), provide a quantitative measure of disease intensity and global neurodegeneration [88].

Despite its high diagnostic accuracy, the invasive nature and perceived clinical burden of lumbar punctures have hindered the widespread adoption of CSF testing in primary care settings. Consequently, the field is undergoing a “plasma revolution.” Utilizing ultra-sensitive immunoassays and mass spectrometry, high-fidelity blood-based biomarkers have emerged. Notably, plasma p-tau217 has demonstrated robust performance, exhibiting high concordance with both CSF assays and PET neuroimaging [89]. With an accuracy exceeding 90% in distinguishing AD from non-AD dementias, plasma p-tau217 is poised to transform primary screening and diagnostic triage [90]. Furthermore, the quantification of the plasma Aβ42/40 ratio provides a highly scalable modality to detect amyloid positivity years prior to overt cognitive decline, addressing a critical bottleneck in therapeutic trial enrolment.

However, a critical translational gap remains regarding the “plasma accessibility illusion.” While plasma biomarkers are universally championed for their potential to democratize AD screening, the ultra-sensitive platforms required to detect these proteins (e.g., single-molecule array technology or advanced mass spectrometry) are currently confined to specialized academic centers and centralized commercial laboratories [91]. Bridging the gap between high-complexity lab requirements and point-of-care, primary clinic accessibility remains a massive infrastructural hurdle for global health equity.

#### 4.1.2. Neuroimaging: Molecular and Structural Insights

While fluid biomarkers excel in detecting systemic pathological presence, molecular neuroimaging via Positron Emission Tomography (PET) is indispensable for mapping the spatial distribution and burden of proteinopathies. Amyloid-PET facilitates the in vivo visualization of fibrillar plaque deposition [92]. However, because amyloid accumulation often plateaus early in the clinical course, its utility lies primarily in confirming the disease trigger and establishing eligibility for anti-amyloid therapeutics. Conversely, Tau-PET tracks the topographical propagation of neurofibrillary tangles. Crucially, the spatiotemporal distribution of tau deposition exhibits a far stronger correlation with synaptic loss and domain-specific cognitive deficits than total amyloid load [93].

#### 4.1.3. Structural Magnetic Resonance Imaging (MRI)

Structural MRI remains the foundational clinical modality for assessing macroscopic neurodegeneration. While PET defines the molecular etiology, MRI quantifies the structural toll. Automated volumetric analyses, particularly measuring hippocampal atrophy and temporoparietal cortical thinning, provide standardized indices of progressive neuronal loss [86]. Beyond gross structural changes, advanced modalities like Diffusion Tensor Imaging (DTI) are increasingly leveraged to detect early microstructural alterations in white matter tracts [94]. These microstructural metrics offer a vital mechanistic link between central neurodegeneration and the peripheral neuropathic vulnerabilities frequently observed in AD populations [95]. Ultimately, the future of AD diagnostics relies on a tiered, multimodal approach: initiating with accessible, high-throughput plasma screening, followed by targeted confirmatory testing via CSF or PET imaging to definitively guide the administration of disease-modifying therapies, as summarized in Table 2.

However, these future perspectives must be critically contextualized within the realities of global clinical practice. While CSF and PET biomarkers are extremely valuable for definitive AD diagnosis, target engagement confirmation, and deep mechanistic understanding, their routine clinical application is severely limited by logistical and clinical constraints. Specifically, broad implementation is hindered by the limited accessibility of specialized medical infrastructure, the inherent invasiveness and patient burden of CSF collection, and the prohibitive financial cost and restricted availability of PET imaging facilities. These barriers underscore the urgency of transitioning toward scalable blood-based screening to democratize early diagnosis [96].

**Table 2:** Comparative diagnostic performance of ad biomarkers [97,98,99].

Biomarker	Modality	Sensitivity	Specificity	Primary Clinical Utility
p-tau217	Plasma	90–96%	85–95%	High-accuracy, non-invasive screening; predicts PET positivity.
Aβ_42/40_ ratio	Plasma	80–85%	75–85%	Early screening, though more sensitive to lab processing than p-tau.
p-tau (181/217)	CSF	90–95%	90–95%	Gold standard for confirming tau pathology and staging.
Aβ_42/40_ ratio	CSF	90–95%	85–90%	Confirms amyloid “positivity” and early-stage deposition.
Amyloid PET	Imaging	90–95%	85–95%	Visualizes spatial plaque load; used for DMT eligibility.
Tau PET	Imaging	85–90%	90–95%	Strongest correlation with cognitive symptoms and decline.
Volumetric MRI	Imaging	75–85%	70–80%	Measures neurodegeneration; non-specific to AD but tracks progression.

Several key analytical takeaways emerge from this comparative performance. Most notably, plasma p-tau217 has effectively reached diagnostic parity with CSF and PET methodologies, representing a transformative leap that enables high-confidence screening in primary care and circumvents cost-prohibitive or invasive initial evaluations [100]. However, while fluid markers indicate the presence of pathology, PET remains indispensable for spatial mapping; it is uniquely required for clinical trials and the safe administration of anti-amyloid monoclonal antibodies to confirm target engagement in vivo [101]. Consequently, the current reliance on PET and CSF for final therapeutic eligibility inevitably restricts access to novel therapies to populations residing near well-resourced, specialized centers. Furthermore, although MRI is highly sensitive to macroscopic neurodegeneration, its specificity for AD is limited because atrophy routinely overlaps with other age-related pathologies like Limbic-predominant age-related TDP-43 encephalopathy (LATE) or vascular dementia [102]. Finally, in both plasma and CSF analyses, utilizing the Aβ42/40 ratio yields significantly higher diagnostic accuracy than absolute Aβ42 concentrations, as the ratio intrinsically normalizes inter-individual variations in baseline amyloid production [103].

### 4.2. PNS: NCS/EMG, Small-Fiber Biopsy, Serologic and Autoimmune Markers

**Figure 4:**
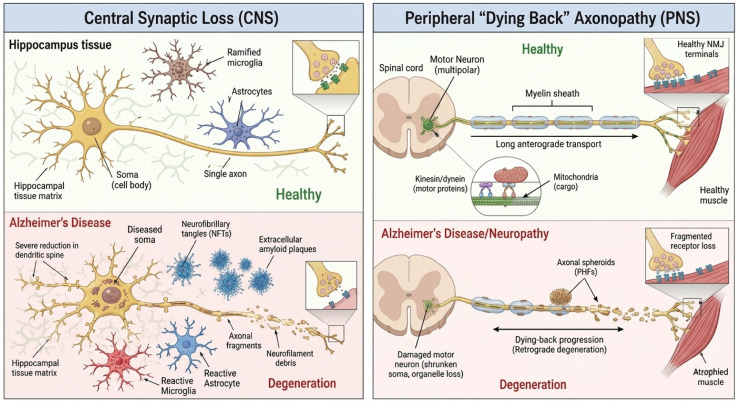
Comparative pathology of central and peripheral neurodegeneration. This schematic contrasts the mechanisms of structural collapse in the central nervous system (CNS) and the peripheral nervous system (PNS). In the CNS (**left panels**), synaptic loss and neurodegeneration are driven by the accumulation of extracellular amyloid plaques and intracellular neurofibrillary tangles (NFTs), exacerbated by the proliferation of reactive microglia and astrocytes. This toxic environment results in a severe reduction in dendritic spines and overall synaptic terminal loss. In the PNS (**right panels**), structural collapse manifests as a retrograde “dying-back” axonopathy driven by metabolic and axonal transport failures, specifically involving kinesin/dynein motor proteins and mitochondrial cargo. This distal-to-proximal progressive damage leads to the accumulation of axonal spheroids, fragmentation of neuromuscular junction (NMJ) receptors, shrinkage of the motor neuron soma, and subsequent muscle atrophy.

While AD is classically defined by central nervous system (CNS) pathology, a robust and expanding body of evidence dictates that the peripheral nervous system (PNS) is concurrently compromised. These peripheral manifestations, frequently presenting as subclinical sensory and motor neuropathies, often precede or parallel cognitive decline [104]. Consequently, assessing the PNS in AD cohorts provides an accessible, in vivo “window” into systemic axonal health, leveraging electrophysiological, morphological, and serological diagnostics to map the true systemic extent of neurodegeneration (Figure 4).

#### 4.2.1. Electrophysiological Assessment: NCS and EMG

Nerve conduction studies (NCS) and electromyography (EMG) remain the standard clinical modalities for evaluating large-fiber peripheral nerve function. In AD populations, these electrophysiological evaluations frequently reveal subclinical or overt sensorimotor polyneuropathies, characterized by attenuated compound muscle action potential (CMAP) and sensory nerve action potential (SNAP) amplitudes [105]. These decrements point toward a predominantly axonal pathophysiological process, aligning with the “dying-back” hypothesis wherein the distal segments of the most metabolically demanding, long-range axons are the first to degenerate secondary to bioenergetic failure [106]**.** Furthermore, EMG can identify chronic denervation and reinnervation patterns within distal musculature. Crucially, these electrophysiological deficits correlate directly with early gait instability and an elevated risk of falls, underscoring the clinical imperative of integrating PNS evaluation into the comprehensive management of AD [107].

#### 4.2.2. Morphological Markers: Small-Fiber Biopsy

Traditional large-fiber assessments are limited by their inability to detect small-fiber neuropathy affecting unmyelinated C-fibers and thinly myelinated Aδ-fibers [108]. Skin biopsy with quantification of Intraepidermal Nerve Fiber Density (IENFD) has emerged as the most validated diagnostic tool for small-fiber neuropathy, boasting a sensitivity of 74–80% and specificity of 65–90% [109]. Because skin biopsies offer a highly accessible, repeatable metric of axonal integrity compared to central imaging, IENFD serves as an emerging potential peripheral proxy for evaluating the target engagement and efficacy of novel neuroprotective therapies [110]. However, the field urgently requires the validation of peripheral biomarkers that can reliably distinguish AD-related changes from normal aging to clarify whether peripheral involvement represents a primary “dying-back” pathology or a secondary effect of central neurodegeneration.

#### 4.2.3. Serologic and Autoimmune Markers

The bidirectional pathophysiological crosstalk between the CNS and PNS is further elucidated through systemic serologic profiling. Chronic, low-grade systemic inflammation—characterized by elevated circulating levels of C-reactive protein (CRP), IL-1β, and TNF-α—is a ubiquitous, shared feature driving both AD progression and peripheral nerve dysfunction [111]. Furthermore, metabolic derangements, such as elevated homocysteine, are frequently observed in AD patients; hyperhomocysteinaemia functions as a potent systemic neurotoxin that independently promotes both central hippocampal atrophy and peripheral axonal degradation [112]. Recent investigations have also begun to interrogate the role of autoimmune markers, noting that a distinct subset of patients exhibits circulating autoantibodies directed against neural antigens, including anti-ganglioside antibodies or those targeting the α-7 nicotinic acetylcholine receptor [113].

### 4.3. Integrative Biomarker Framework Linking Mechanisms to Phenotype

The inherent pathophysiological complexity of AD necessitates a paradigm shift from a monolithic, symptom-based diagnostic model to a dynamic, multi-modal biological framework. The NIA-AA ATN framework provided the initial scaffolding for defining AD biologically in vivo [86]. However, critical evaluations of this model note its limitations in capturing the systemic heterogeneity of the disease. Recognizing this, recent iterations have formally expanded to include inflammatory (I) indices, reflecting a vital shift toward an ATN(I) or broader ATN(X) framework capable of capturing systemic immune dysregulation [96,114]. To fully bridge central neurodegeneration with peripheral nerve dysfunction, this must be conceptually expanded further to an integrative ATN(I)(M) model, incorporating metabolic (M) markers.

The standard ATN profile establishes the core biological presence of AD. Amyloid positivity (A+), determined via PET or plasma ratios, represents the initiating biological state, while Tau positivity (T+) signifies the active disease stage and correlates with classic amnestic phenotypes [86]. Neurodegeneration (N+), marked by elevated NfL or structural atrophy, reflects cumulative downstream injury. Consequently, an A+T+N+ profile strongly predicts the classic trajectory of rapid-onset, multidomain dementia, whereas an A+T-N- profile indicates early pathologic change, delineating a critical window for preventative intervention [96].

Beyond the core proteins, the trajectory of cognitive decline is heavily modulated by the innate immune response (the Inflammatory Axis). Fluid biomarkers such as plasma glial fibrillary acidic protein (GFAP) and soluble TREM2 (sTREM2) serve as proxies for reactive astrocytosis and microglial activation [115]. Patients presenting with elevated inflammatory markers (I+) alongside core AD pathology frequently exhibit a “rapid progressor” clinical phenotype, as unresolved neuroinflammation accelerates synaptic pruning [116]. Integrating the Metabolic Axis (M)—such as indices of insulin resistance (Homeostatic Model Assessment of Insulin Resistance (HOMA-IR)) and lipidomic profiles—further identifies patients with a “mixed” phenotype. Clinically, an ATN+ Metabolic (M+) profile manifests early as gait disturbances, motor slowing, and small-fiber peripheral neuropathy [117]. By evolving from a binary diagnostic label to a multidimensional narrative ATN(I)(M) profile, clinicians can effectively deconstruct the heterogeneous presentation of AD to guide highly personalized interventions.

## 5. Phenotype–Mechanism Correlation

### 5.1. Cognitive and Neuropathic Phenotypes: The Clinical Spectrum

The clinical manifestation of Alzheimer’s disease (AD) and its associated neuropathies can no longer be viewed as a monolithic, central nervous system (CNS)-isolated syndrome. Rather, it represents a continuous, multisystem spectrum of neurological dysfunction. As outlined in recently updated diagnostic frameworks [86,96], accurate patient stratification via this biological continuum is the requisite foundation for selecting mechanism-driven, personalized therapies. The distinct trajectories and differential diagnoses of these AD clinical phenotypes are synthesized in Table 3.

#### 5.1.1. The Amnestic-Predominant Phenotype (Classic CNS)

This classic phenotype is primarily initiated by early Aβ deposition (A+), measurable via Amyloid-PET Standardized Uptake Value Ratio (SUVr) or CSF Aβ42/40 ratios, often showing initial accumulation within the default mode network [124]. This initiating pathology is subsequently followed by the propagation of tau tangles (T+) into the entorhinal cortex and hippocampus, readily identifiable through Tau-PET or advanced fluid assays like plasma/CSF p-tau217 [93]. Radiologically, this cascade correlates with disproportionate medial temporal atrophy on structural MRI [125]. Clinically, it manifests as profound episodic memory impairment and progressive disorientation, which remains the hallmark of typical AD progression.

#### 5.1.2. Non-Amnestic and Atypical Variants

Phenotypes such as Posterior Cortical Atrophy (PCA) or Logopenic variant Primary Progressive Aphasia (lvPPA) critically demonstrate that the topographical distribution of proteinopathies—rather than the proteins themselves—dictates the clinical outcome [119]. In these atypical variants, Tau-PET frequently bypasses the hippocampus, showing intense uptake in specific cortical hubs such as the parieto-occipital or left temporal lobes. Despite divergent clinical presentations, these variants remain biologically anchored in the AD continuum, confirmed via A+/T+ fluid biomarkers [93].

#### 5.1.3. The Peripheral Neuropathic Phenotype (PNS Focus)

Historically underrecognized, the neuropathic phenotype highlights the systemic etiology of AD. Clinically, it often manifests early as distal sensory loss, gait instability, and autonomic dysfunction. This phenotype is deeply linked to systemic metabolic failure, often framed as “Type 3 Diabetes” [8], where cerebral insulin resistance creates a toxic metabolic environment detectable early via global hypometabolism on Fluorodeoxyglucose (FDG)-PET. However, a fierce controversy persists in the field regarding the true origin of this phenotype. Traditionalists argue that peripheral neuropathy in AD cohorts is merely an epiphenomenon—a secondary artifact driven by parallel, age-related metabolic decline and highly prevalent, undiagnosed type 2 diabetes [8]. Conversely, systemic theorists contend that peripheral degradation is a fundamental, direct consequence of AD proteinopathies propagating along the neuro-axis [126]. Resolving this controversy is critical, as it determines whether peripheral nerves are distinct therapeutic targets or merely collateral damage.

#### 5.1.4. The Mixed Phenotype (CNS-PNS Crosstalk)

This represents the most complex and rapidly progressive clinical presentation, where global cognitive decline may correlate with peripheral sensory–motor deficits [106]. Evidence suggests a systemic “inflammatory echo” significantly accelerates the rate of pan-neuronal degeneration [127]. High levels of plasma Neurofilament Light chain (NfL) serve as an important unified biomarker; because NfL is a structural component of the axonal cytoskeleton, it acts as an integrated measure of both central and peripheral axonal death [123].

### 5.2. CNS–PNS Mechanistic Interactions: The Biological Bridge

Moving from clinical manifestations to their molecular underpinnings requires abandoning the outdated, monolithic amyloid cascade hypothesis. The clinical heterogeneity of AD can only be understood through precise phenotype–mechanism correlations that view the body as an integrated system. A critical advancement in this domain is the recognition of a bidirectional “biological bridge” between the CNS and the peripheral nervous system (PNS) [128]. While the classical amnestic phenotype correlates neatly with the spatiotemporal spread of tau pathology, patients exhibiting the “mixed” phenotype reveal a different underlying reality driven by two primary shared vulnerabilities: systemic inflammatory crosstalk and bioenergetic failure [127].

Chronic activation of the innate immune system—specifically, pathological microglial activation in the brain and macrophage infiltration in peripheral nerves—creates a systemic cytokine storm. This circulating inflammatory burden degrades the structural integrity of both the blood–brain barrier (BBB) and the blood-nerve barrier (BNB) [129]. Consequently, toxic oligomers and inflammatory mediators circulate freely between compartments, driving synergistic degenerative processes across the entire neuro-axis. Concurrently, the central and peripheral nervous systems share a critical reliance on massive, uninterrupted energy supplies. Systemic metabolic failures trigger a devastating dual-compartment energy crisis [130]. This glucose hypometabolism simultaneously starves the hippocampus—impairing synaptic plasticity—and disrupts the highly energy-intensive, long-distance axonal transport essential for PNS maintenance, leading directly to distal “dying back” neuropathies [131,132,133].

**Figure 5:**
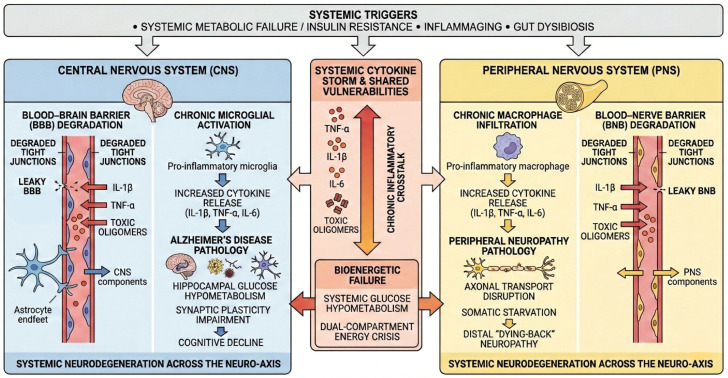
The CNS–PNS biological bridge. Systemic triggers drive a shared cytokine storm and bioenergetic crisis, leading to the parallel degradation of the blood–brain barrier (BBB) and blood–nerve barrier (BNB). This interconnected neuroinflammatory cascade precipitates synergistic neurodegeneration across both the central and peripheral nervous systems.

To conceptualize these interconnected pathological events, Figure 5 provides a comprehensive visual summary of this CNS–PNS “biological bridge.” The schematic illustrates how shared systemic triggers—such as metabolic failure, inflammaging, and gut dysbiosis—converge to initiate a massive systemic cytokine storm and a dual-compartment bioenergetic crisis. This central hub of systemic vulnerability subsequently drives the parallel degradation of the blood–brain barrier (BBB) and blood–nerve barrier (BNB). By mapping the concurrent activation of central microglia and peripheral macrophages alongside the infiltration of toxic oligomers, the figure highlights how these shared mechanisms result in both hippocampal starvation and distal axonal transport disruption, ultimately culminating in synergistic neurodegeneration across the entire neuro-axis.

### 5.3. Translational Relevance for Patient Stratification

The historical conceptualisation of AD as a homogenous clinic-pathological entity has been definitively superseded by a precision medicine paradigm. Recognizing and mapping phenotype–mechanism correlations is now the absolute cornerstone of modern translational neuroscience. The clinical reality is that the rate of cognitive decline is heavily dictated by concurrent co-pathologies, including α-synuclein, TDP-43, and cerebrovascular disease, rendering the concept of “pure AD” virtually obsolete in aging populations [32].

Yet, a glaring translational controversy lies in the stark disconnect between this recognized phenotypic heterogeneity and contemporary clinical trial design. While precision medicine dictates targeting specific mechanisms, current pivotal Phase 3 trials for anti-amyloid monoclonal antibodies—such as the Clarity AD trial for lecanemab (NCT03887455) and the TRAILBLAZER-ALZ 2 trial for donanemab (NCT04437511)—rigidly enforce homogeneous inclusion criteria [134]. By aggressively screening out patients with significant vascular, metabolic, or mixed pathologies, the field systematically excludes the vast majority of patients who exhibit complex, metabolically driven phenotypes. Consequently, we risk approving therapeutics whose efficacy and safety profiles are entirely unknown for the average, real-world AD patient. Integrating detailed phenotypic data with specific biomarker profiles ensures that individuals whose phenotypes are heavily driven by systemic inflammatory or metabolic dysfunctions are directed toward targeted interventions, avoiding inappropriate funneling into broadly applied anti-amyloid monotherapies.

## 6. Therapeutics and Translational Applications

### 6.1. Alzheimer’s: Symptomatic and Disease-Modifying Therapies

**Figure 6:**
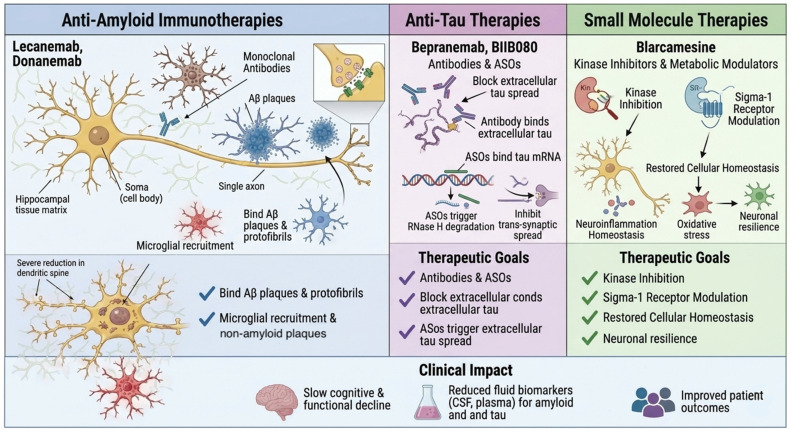
Integrated mechanisms of Alzheimer’s disease modifying therapies. This schematic outline three primary therapeutic strategies aimed at altering the progressive course of Alzheimer’s disease. Anti-amyloid immunotherapies (e.g., lecanemab, donanemab) utilize monoclonal antibodies to bind Aβ plaques and protofibrils, facilitating plaque clearance via microglial recruitment and phagocytosis. Anti-tau therapies employ distinct mechanisms to halt tau pathology, including antisense oligonucleotides (ASOs) that trigger targeted mRNA degradation to reduce tau synthesis, and monoclonal antibodies that block the extracellular, trans-synaptic spread of tau. Lastly, small molecule therapies (e.g., blarcamesine) modulate intracellular signaling through kinase inhibition and Sigma-1 receptor activation, restoring cellular homeostasis by mitigating oxidative stress and neuroinflammation. Together, these targeted interventions aim to reduce fluid biomarkers (CSF and plasma), slow cognitive and functional decline, and ultimately improve patient outcomes.

The therapeutic landscape for AD is finally beginning to reflect the biological complexity outlined in previous chapters. The paradigm is shifting from purely symptomatic management to disease-modifying therapies (DMTs) that attempt to alter underlying pathophysiological trajectories. Treatment strategies are currently stratified into symptomatic agents and DMTs, with the latter aggressively targeting Aβ clearance, tau propagation, and broader systemic metabolic pathways, as depicted in Figure 6.

#### 6.1.1. Symptomatic Therapies

For decades, the standard of care has been fundamentally palliative. Cholinesterase inhibitors (donepezil, rivastigmine, galantamine) support failing memory circuits, while memantine mitigates glutamate excitotoxicity. While these agents offer a brief window of symptomatic stabilization, they are entirely incapable of arresting structural axonal loss or resolving the underlying metabolic crises driving the neurodegeneration [135].

#### 6.1.2. Disease-Modifying Therapies (DMTs)

Monoclonal antibodies targeting Aβ represent the first major breakthrough in structural DMTs. Lecanemab and donanemab have achieved regulatory approval by demonstrating a 27–35% slowing of cognitive decline in early, amyloid-confirmed AD [19,20]. However, this class is at the center of a fierce clinical controversy: the debate over statistical versus clinical significance. Critics argue that a 27% slowing on an 18-point cognitive scale equates to a fraction of a point—a difference often imperceptible to patients and caregivers [19,20,136]. Furthermore, their utility is severely limited by a substantial risk of Amyloid-Related Imaging Abnormalities (ARIA), effectively excluding patients with the mixed pathologies or cerebrovascular fragility most common in real-world aging populations [136].

Because tau pathology correlates much more closely with cognitive decline than amyloid, it is a higher-value target. After early clinical trials utilizing extracellular monoclonal antibodies yielded disappointing results [137], the field is pivoting toward intracellular interventions. Antisense Oligonucleotides (ASOs), which degrade tau mRNA to fundamentally lower total tau protein production, have shown significant promise in early-phase trials [138]. Finally, small molecule therapies offer oral bioavailability and superior blood–brain barrier (BBB) penetration. Compounds like hydromethylthionine mesylate (LMTM) attempt to dissolve tau aggregates, while kinase inhibitors target Glycogen synthase kinase-3β (GSK-3β) or Cyclin-dependent kinase 5 (CDK5). Crucially, addressing systemic failure requires non-amyloid approaches; agents like blarcamesine aim to restore cellular homeostasis and dampen widespread neuroinflammation [139].

### 6.2. Neuropathies: Immune Modulation, Metabolic Therapy, and Gene-Targeted Approaches

**Figure 7:**
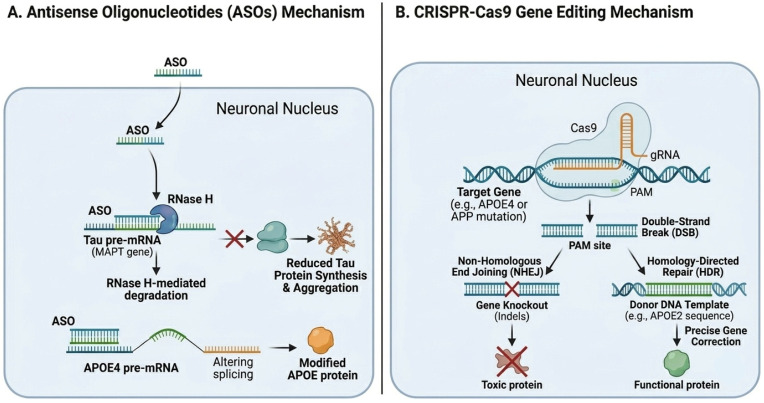
RNA and DNA-targeted therapeutic modalities. This diagram illustrates two distinct gene-based strategies for treating neurodegenerative diseases. (**A**) Post-transcriptional gene modification via Antisense Oligonucleotides (ASOs), demonstrating targeted pre-mRNA degradation mediated by RNase H (e.g., reducing tau synthesis) and alternative splicing modulation (e.g., modifying APOE4) [138,140,141]. (**B**) Permanent genomic editing via CRISPR-Cas9, showing how targeted double-strand breaks lead to either gene knockout of toxic proteins via Non-Homologous End Joining (NHEJ) or precise gene correction to functional proteins via Homology-Directed Repair (HDR) using a donor DNA template [142,143].

To effectively alter the disease course, therapeutics must treat AD and its associated peripheral neuropathies as interconnected disorders. Achieving this requires a comprehensive, multi-modal strategy encompassing systemic immune modulation, metabolic restoration, and advanced gene-targeted interventions. The specific mechanisms driving these emerging molecular approaches—including the RNA- and DNA-targeted therapies illustrated subsequently in Figure 7—will be explored in detail throughout the following subsections.

Neuroinflammation is an active driver bridging central cognitive decline and peripheral nerve damage [144]. Through a compromised BBB, central cytokines (TNF-α, IL-1β) spill into the periphery, while systemic signals recruit peripheral macrophages into the CNS [145,146]. Modern targeted therapies aim at the NOD-like receptor family pyrin domain containing 3 (NLRP3) inflammasome—a universal trigger for this cytokine storm—to simultaneously extinguish the inflammatory response in both central networks and peripheral nerve beds [147].

Furthermore, metabolic therapy directly addresses the profound, localized insulin resistance in the AD brain, a phenotype that structurally mirrors the metabolic starvation seen in diabetic peripheral neuropathy. Glucagon-like peptide-1 (GLP-1) receptor agonists represent a massive breakthrough in neuroprotective strategy; these agents cross the BBB to enhance mitochondrial biogenesis and resensitize insulin signaling [148]. Despite immense epidemiological promise, skeptics question whether GLP-1 agonists exert direct neuroprotective effects on the brain, or if their cognitive benefits are merely secondary artifacts of systemic weight loss and improved peripheral glycemic control. Ongoing Phase 3 trials must definitively untangle this systemic versus central efficacy.

Gene-targeted approaches intervene directly at the genetic source. As illustrated in Figure 7, ASOs facilitate post-transcriptional gene silencing by recruiting RNase H to degrade target pre-mRNA (e.g., microtubule-associated protein tau (MAPT)), halting the synthesis of toxic tau proteins before aggregation occurs [138]. Alternatively, clustered regularly interspaced short palindromic repeats (CRISPR)-Cas9 enables permanent genomic modification via guide RNA (gRNA)-directed double-strand breaks, offering a theoretical pathway to replace a high-risk apolipoprotein E (APOE) ε4 sequence with the protective APOE ε2 variant in vivo [149]. Yet, the severe ethical and safety risks of irreversible genomic editing in the aging brain—combined with the high potential for off-target mutations and the immense challenge of safe viral vector delivery across the BBB—currently keeps CRISPR confined to theoretical models rather than late-onset AD applications.

### 6.3. Mechanism-Driven Therapy Selection and Current Evidence

The advent of early-stage DMTs has necessitated a transition to mechanism-driven precision medicine, where therapeutic selection is predicated on the dominant biological drivers identified in vivo via the patient’s specific biomarker profile. For amyloid-positive (A+) patients, clearing the initiating trigger is paramount; lecanemab and donanemab selectively bind and clear amyloid via microglial phagocytosis, though evidence confirms this aggressive debulking is clinically beneficial only in the earliest stages, before tau propagation becomes irreversible [7,19].

Conversely, for tau-positive (T+) patients, clearing amyloid is insufficient to stop cognitive decline. Targeting the tau axis is required to stop pathological spread; a Phase 1b trial of the tau-targeting ASO BIIB080 demonstrated a remarkable, dose-dependent reduction in CSF total tau, offering a critical advance for phenotypes actively driven by self-propagating pathology [150]. Furthermore, patients exhibiting a combined central hypometabolism and peripheral neuropathy phenotype require targeted interventions along the Metabolic Axis. While definitive Phase 3 AD trials for GLP-1 agonists are ongoing, post hoc analyses of cardiovascular trials indicate a significantly reduced incidence of dementia in long-term users [151]. Finally, for patients with a high systemic inflammatory load (elevated GFAP or sTREM2), modulating the innate immune response is essential. Small-molecule inhibitors of NLRP3 hold the distinct promise of simultaneously arresting the central cytokine storm and mitigating neuropathic pain pathways in the PNS [152].

### 6.4. The Future: Sequential Combination Therapy

**Figure 8:**
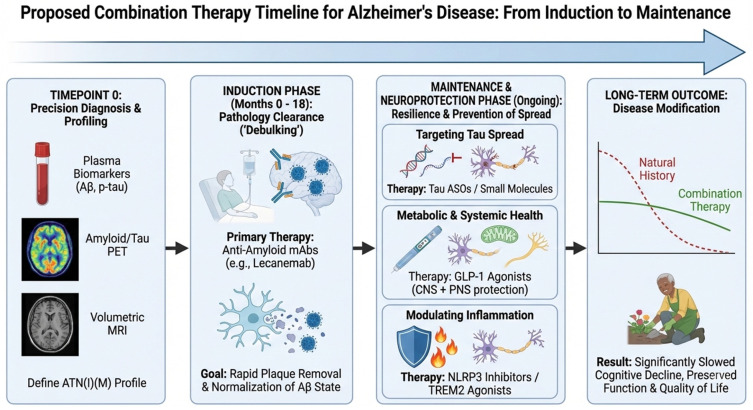
Proposed sequential combination therapy timeline for Alzheimer’s disease. This model advocates for a “Treat-and-Maintain” strategy, utilizing an initial induction phase (anti-amyloid antibodies to debulk plaques) followed by a lifelong maintenance phase (tau-targeting ASOs, GLP-1 agonists, and immunomodulators) to ensure systemic neuronal resilience.

Ultimately, the future of neurodegenerative treatment lies in combination therapy. A patient will likely receive an intensive anti-amyloid induction regimen to rapidly “clean” the brain of initiating plaques, followed by a lifelong metabolic and inflammatory maintenance therapy (e.g., GLP-1 agonists) to ensure systemic neuronal resilience, supplemented by an anti-tau agent to prevent further cortical spread (Figure 8).

However, a massive regulatory and financial controversy shadows this future. Currently, the Food and Drug Administration (FDA) and the European Medicines Agency (EMA) require isolated efficacy data for drug approval [153]. This requirement makes it logistically and financially unfeasible for pharmaceutical companies to test multiple patented, high-cost biologicals in combination trials. Until regulatory frameworks adapt to an oncology-style combination model, this optimal therapeutic strategy remains paralyzed.

## 7. Clinical Trials and Critical Appraisal

### 7.1. Overview of Recent Pivotal Trial

The recent success of pivotal Phase 3 clinical trials targeting the amyloid cascade has definitively validated the use of neuroimaging and fluid markers as active pharmacodynamic indicators of therapeutic efficacy [19,20,86].

#### 7.1.1. Pivotal Trials and Disease-Modifying Therapies (DMTs)

The successful deployment of monoclonal antibodies has revolutionized the treatment paradigm by demonstrating that the substantial, rapid clearance of amyloid plaques correlates directly with a slowing of clinical decline. A summary of these pivotal Phase 3 trials is presented in Table 4.

Specifically, the paradigm-shifting Clarity AD trial for lecanemab met its primary endpoint, demonstrating a 27% reduction in clinical and cognitive decline over 18 months in patients with early AD [19]. Beyond the clinical outcomes, the trial firmly established Amyloid-PET as a robust pharmacodynamic biomarker, confirming a time-dependent, quantitative reduction in plaque burden that served as the foundational secondary evidence for its full regulatory approval. Similarly, the TRAILBLAZER-ALZ 2 trial for donanemab introduced a highly innovative “start–stop” dosing regimen, where aggressive intravenous treatment was completely transitioned to placebo once participants achieved an amyloid-negative status on PET imaging [20]. Crucially for precision medicine, the greatest clinical benefit—a 35% reduction in decline measured via the integrated Alzheimer’s Disease Rating Scale (iADRS)—was observed in patients strictly stratified with “low-to-medium” baseline tau burden, positioning Tau-PET as a mandatory tool for treatment selection [20]. Historically, the EMERGE and ENGAGE trials for aducanumab demonstrated that high-dose monoclonal therapy could successfully reduce brain amyloid levels, leading to a highly controversial accelerated FDA approval based purely on a surrogate biological endpoint [154]. However, due to severe safety profiles and mixed clinical efficacy, the drug was commercially discontinued in 2024, yielding the landscape to the superior safety profiles of lecanemab and donanemab [155].

#### 7.1.2. Critical Appraisal and the Role of Imaging

The clinical appraisal of these DMTs emphasizes that biomarker-confirmed pathology is now an absolute prerequisite before initiating therapy. However, several profound translational challenges remain [156]. The most significant, dose-limiting safety concern across all anti-amyloid trials is the development of Amyloid-Related Imaging Abnormalities, manifesting as either edema (ARIA-E) or haemosiderin deposition (ARIA-H). This highlights the underlying vascular fragility of the AD brain and mandates rigorous, ongoing MRI monitoring protocols to detect subclinical complications before they become fatal [157]. Furthermore, emerging evidence from donanemab clearly suggests that the “window of opportunity” for amyloid-clearing therapies is entirely dictated by the baseline tau burden, elevating Tau-PET from a simple diagnostic aid to an essential prognostic gatekeeper [20]. Finally, an intense point of ongoing critical appraisal revolves around whether the statistically significant slowing of decline observed in these trials actually achieves the Minimal Clinically Important Difference (MCID) for individual patients. While the biological effect of plaque clearance is undeniable, the true clinical priority remains extending the period of functional independence, rather than merely altering a cognitive test score by a fraction of a point.

### 7.2. Biomarker-Driven Endpoints and Surrogate Validation

The validation of biological markers as surrogate clinical endpoints has fundamentally and irreversibly altered the regulatory landscape of AD therapeutics. The modern paradigm demands the integration of high-resolution neuroimaging and advanced fluid assays to provide objective in vivo evidence of both target engagement and downstream disease modification.

#### 7.2.1. The Radiological Concept of Surrogate Endpoints

A surrogate endpoint is a biological marker intended to substitute for a direct clinical outcome. The rapid, quantitative reduction in brain Aβ plaques—measured via Amyloid-PET Standardized Uptake Value ratio (SUVr) analysis—has been successfully utilized as a surrogate marker “reasonably likely” to predict cognitive preservation [158]. However, a fierce regulatory controversy persists regarding this precedent. Critics argue that regulatory willingness to approve drugs based primarily on plaque clearance fundamentally lowers the bar for clinical efficacy, potentially allowing costly drugs to reach the market that successfully “treat the scan” but fail to meaningfully treat the patient [156].

#### 7.2.2. Pharmacodynamic Monitoring (Integrating Image and Fluid)

The role of the neuroradiologist is increasingly intertwined with advanced fluid biomarker analysis to holistically validate the true biological effect of a drug. A critical radiological observation during anti-amyloid therapy is the initial, paradoxical decrease in cortical volume following massive plaque clearance. This “pseudo-atrophy” effect—likely driven by the resolution of neuroinflammation and the physical removal of amyloid bulk—must be carefully correlated with clinical stability to avoid a catastrophic misinterpretation of accelerated disease progression [125]. Consequently, radiological findings must be robustly complemented by systemic markers of neuroaxonal injury. A simultaneous reduction in brain amyloid on PET paired with a stabilization or drop in plasma Neurofilament Light Chain (NfL) and p-tau217 provides the ultimate multi-modal validation of genuine neuroprotection.

#### 7.2.3. Safety Monitoring (The Radiological Responsibility)

Ultimately, the clinical viability of these DMTs depends entirely on the radiologist’s ability to actively manage and mitigate ARIA. Absolute mastery of fluid-attenuated inversion recovery (FLAIR) and T2* gradient-recalled echo (GRE) MRI sequences is a mandatory prerequisite for safely navigating the precision medicine era [125,159].

### 7.3. Clinical Implementation

The clinical implementation of DMTs has abruptly transitioned from a theoretical ideal to a logistical and ethical crucible that fundamentally challenges current healthcare infrastructures. A primary limitation of the precision medicine paradigm is the profound disparity in access to advanced neuroimaging and fluid assays. The strict reliance on PET imaging creates a severe diagnostic bottleneck. Ethically, this paradigm risks transforming Alzheimer’s disease modification into a privilege exclusive to well-resourced tertiary academic centers, drastically exacerbating global health inequalities [160].

Furthermore, the detection and management of ARIA present a profound ethical dilemma. The controversy lies in the fundamental paradox of modern DMTs: treating a mildly symptomatic, functionally independent patient with a biologic agent that carries a distinct risk of causing life-threatening iatrogenic complications [136,157]. This places the neuroradiologist at the center of the ethical debate and highlights an ongoing controversy regarding the true clinical value of these therapies. Health economists and clinicians alike debate whether the staggering financial cost of the drug, paired with the massive resource burden of serial safety MRIs and PET scans, is justified by the marginal gains in a patient’s daily quality of life [161].

Finally, the shift toward a purely biological definition of AD inherently allows for diagnosis in the preclinical, entirely asymptomatic stage. This introduces a profound ethical minefield regarding the psychological trauma of delivering a molecular diagnosis of a terminal neurodegenerative disease to a cognitively normal individual, often years before symptoms manifest [162]. This controversial reality demands a massive shift toward robust, mandatory psychological counseling frameworks—particularly because early-stage preventative interventions remain largely investigational, leaving asymptomatic patients with a devastating diagnosis but no immediately actionable cure [163].

## 8. Future Directions and Translational Priorities

### 8.1. Emerging Molecular Targets and Pathways

**Figure 9:**
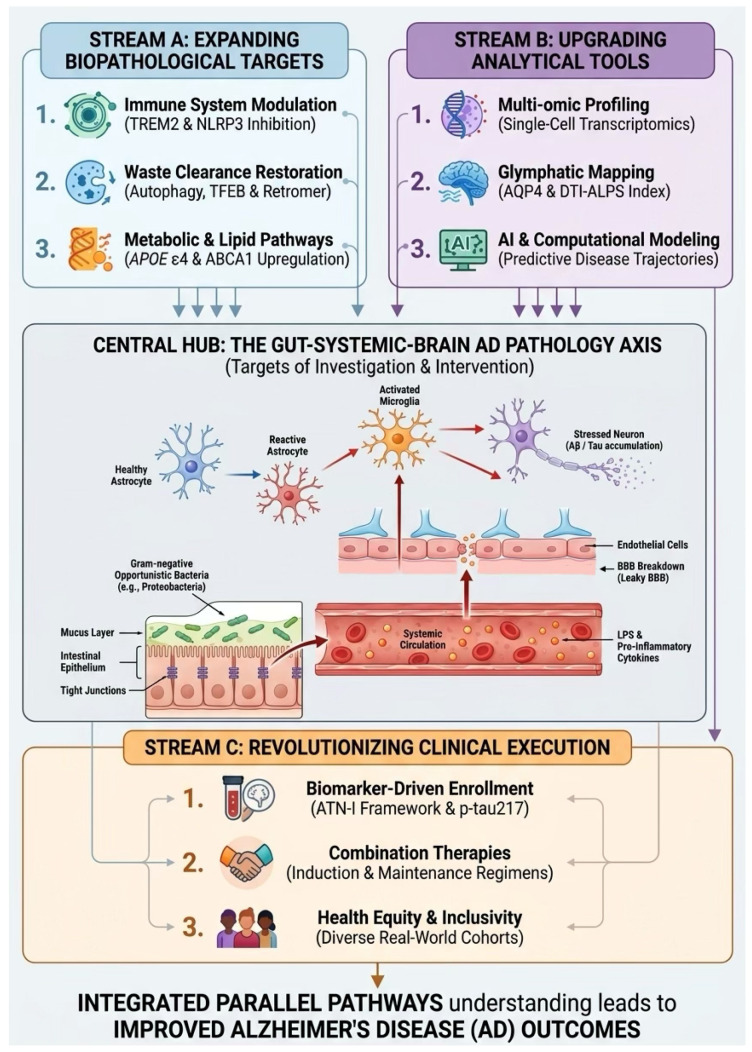
Strategic roadmap for translational priorities in AD research. This framework outlines the necessary evolution of AD therapeutics from isolated protein clearance to systemic, precision-medicine interventions. Addressing traditional sequential models, this paradigm presents three concurrent, parallel research streams: expanding biopathological targets (Stream A), upgrading analytical tools (Stream B), and revolutionizing clinical execution (Stream C). Streams A and B operate simultaneously to deepen the mechanistic understanding and therapeutic targeting of the core Gut–Systemic–Brain AD Pathology Axis (Central Hub). These integrated, multi-modal insights subsequently drive Stream C, facilitating biomarker-driven enrollment, targeted combination therapies, and inclusive, real-world clinical trial designs to ultimately improve patient outcomes.

The therapeutic landscape for AD is finally—and rapidly—expanding beyond the restrictive confines of the traditional amyloid cascade hypothesis to embrace the multifactorial, systemic nature of the disease. Current translational research is heavily pivoting toward emerging molecular targets that capture the systems-level collapse of the AD brain. As outlined in the strategic roadmap in Figure 9, therapeutic development must move beyond isolated protein clearance to actively address neuroinflammation, restore endolysosomal waste clearance, and correct lipid metabolism deficits [135].

#### 8.1.1. Neuroinflammation and Microglial Modulation

Chronic innate immune activation is now recognized as a central, independent driver of AD pathogenesis. Modulating microglial surface receptors, particularly TREM2, has emerged as a critical therapeutic target. Enhancing TREM2 signaling aims to forcefully shift microglia from a pro-inflammatory state back into a reparative phenotype capable of efficient plaque phagocytosis [164]. Concurrently, targeted inhibition of the NLRP3 inflammasome is being explored to block the downstream cytokine storm [152]. However, a major field controversy complicates this approach: the “double-edged sword” of microglial activation. While enhancing TREM2 might aid in clearing plaques during preclinical stages, hyper-activating microglia in later symptomatic stages could inadvertently accelerate synaptic pruning and neurotoxic damage [165]. Determining the precise temporal window for immune agonism versus antagonism remains a fiercely debated, unresolved challenge.

#### 8.1.2. Endolysosomal and Autophagic Pathways

The progressive accumulation of toxic protein aggregates signifies a catastrophic failure of the neuron’s fundamental clearance mechanisms. The autophagy-lysosome pathway is a high-priority target for restoring proteostasis. Therapeutic strategies are actively targeting TFEB (Transcription Factor EB), the master regulator of lysosomal biogenesis, to upregulate autophagic flux [166]. Additionally, pharmacological stabilization of the retromer complex is being investigated to correct the pathogenic misrouting of the amyloid precursor protein (APP) [167,168].

#### 8.1.3. Lipid Metabolism

Given the profound genetic risk conferred by the APOE ε4 allele, modulating systemic and cerebral lipid transport is a critical avenue for intervention. Targets such as ABCA1, which facilitates the lipidation of APOE, are being aggressively pursued to restore cholesterol homeostasis, enhance compromised blood–brain barrier integrity, and promote synaptic repair [169].

### 8.2. Multi-Omic and Computational Approaches

The pathophysiological landscape of AD is increasingly understood as a systems-level collapse of cellular and network integrity. Capturing this complexity requires upgrading our analytical tools by integrating multi-omic profiling (e.g., single-cell transcriptomics) with advanced computational modeling [170,171].

#### 8.2.1. Multi-Omic Profiling of Network Vulnerability

Single-cell transcriptomics and spatially resolved proteomics have fundamentally advanced our understanding of AD, mapping disruptions at the myelin–axon interface and localized metabolic failures within vulnerable white matter tracts [172]. Simultaneously, transcriptomic profiling reveals how chronic neuroinflammation forces astrocytes into reactive states that mislocalize AQP4 water channels [173]. This stalls glymphatic fluid exchange, creating a toxic extracellular microenvironment that accelerates adjacent axonal dying-back.

#### 8.2.2. Computational Modeling and Imaging Integration

Translating these molecular insights into clinical utility requires sophisticated computational frameworks. Artificial intelligence and machine learning algorithms are now being deployed to integrate vast multi-omic datasets with advanced, non-invasive neuroimaging. For instance, computational models utilizing Diffusion Tensor Imaging alongside the DTI-ALPS (Diffusion Tensor Image Analysis along the Perivascular Space) index allow researchers to actively map glymphatic failure via AQP4 channels and correlate fluid dynamics directly with axonal microstructural degradation [174].

### 8.3. Recommendations for Translational and Clinical Research

To accelerate the development of robust, disease-modifying therapies, future research frameworks must revolutionize clinical execution by prioritizing biomarker integration, target diversification, and cohort inclusivity. Clinical trials must permanently transition away from symptom-based enrolment toward strictly biomarker-driven, preclinical, or early-symptomatic cohorts utilizing the expanded ATN(I) framework. The rigorous validation of scalable fluid markers (particularly plasma p-tau217 and GFAP) will democratize trial access and enable real-time tracking of target engagement [175]. Crucially, this biomarker integration must move beyond strictly neurocentric models. To achieve truly comprehensive trial enrollment, current frameworks must be further expanded to include peripheral and vascular metrics. The integration of emerging precision diagnostics, such as sPDGFRβ for detecting early blood–brain barrier (BBB) breakdown and pericyte injury [45], provides a critical opportunity to stratify patients based on their specific systemic and neurovascular profiles. Identifying these early vascular deficits before irreversible network disconnection occurs will allow researchers to target specific patient subsets with precisely tailored interventions. Furthermore, trial infrastructures must evolve to support adaptive designs capable of evaluating sequential combination therapies. Testing a potent anti-amyloid “induction” phase followed by a neuroprotective or tau-targeting “maintenance” regimen is biologically essential to address the multifactorial nature of the disease.

A glaring imperative for future clinical research is the inclusive enrolment of underrepresented racial, ethnic, and socioeconomic populations. Historically homogeneous trial cohorts severely compromise the generalizability of safety and efficacy data. This represents a profound scientific and ethical controversy: approving biologicals based on cohorts that fundamentally fail to reflect the demographics actually bearing the burden of the disease. For context, older Black and Hispanic populations in the United States are approximately 2 times and 1.5 times more likely to develop AD than older White populations, respectively [11]. Despite this, they are chronically excluded from pivotal data. In the global Phase 3 Clarity AD trial for lecanemab (1795 participants), only 47 participants (2.5%) were Black, and 215 (12%) were Hispanic [19]. Within the U.S.-specific enrolment for that trial, Black participants accounted for merely 4.5%, despite comprising roughly 14% of the U.S. population and over 20% of the U.S. AD demographic [11]. Researchers must aggressively leverage decentralized trial methodologies, digital cognitive phenotyping, and community-based participatory research to close this gap and ensure emerging therapies are safe and effective for all populations [120].

### 8.4. Holistic Disease Management and Lifestyle Interventions

Beyond molecular and pharmacological targets, a comprehensive disease management strategy must incorporate holistic, non-pharmacological lifestyle interventions. Physical activity has emerged as a potent therapeutic consideration, demonstrating significant potential to improve underlying metabolic mechanisms, reduce systemic oxidative stress, and enhance the overall psychological well-being of patients [176]. Similarly, targeted dietary interventions and nutritional modifications play a profound role in altering AD trajectories. These dietary shifts are particularly vital for their direct modulation of the microbiota–gut–brain axis, helping to restore microbial balance, reduce intestinal hyperpermeability, and mitigate systemic inflammaging [177,178]. Finally, the emerging branch of sleep medicine offers critical therapeutic value; given sleep’s fundamental role in facilitating glymphatic waste clearance and maintaining cognitive homeostasis, targeted sleep interventions are essential for slowing AD progression [179]. Ultimately, integrating these clinically relevant lifestyle and metabolic interventions alongside advanced molecular pharmacotherapies will be vital for achieving truly complete, multidimensional disease management.

## 9. Conclusions

### 9.1. Synthesis of Mechanistic, Translational, and Clinical Insights

The era of viewing AD as a localized, intractable proteinopathy has definitively ended. As synthesized in this review, the advent of anti-amyloid disease-modifying therapies validates plaque clearance as a critical pharmacological target, yet simultaneously exposes the severe limitations of monotherapy. Here, the field confronts its most glaring translational controversy: the juxtaposition of undeniable biological target engagement (plaque clearance) against stubbornly marginal clinical cognitive gains and the profound radiological burden of Amyloid-Related Imaging Abnormalities (ARIA). This underscores a fundamental truth: amyloid clearance is merely an induction phase, not a standalone cure.

Mechanistically, AD must now be aggressively targeted as a systemic collapse of the central and peripheral nervous system axis, heavily driven by chronic neuroinflammation, endolysosomal failure, and metabolic starvation. Translating these complex mechanistic insights into real-world clinical utility requires an uncompromising commitment to the expanded ATN(I) biomarker framework. We must strictly utilize advanced neuroimaging and scalable fluid assays (e.g., plasma p-tau217) to guide precision interventions long before irreversible neurodegeneration occurs. Ultimately, the future of AD management dictates a highly targeted, sequential combination approach—pairing early amyloid “debulking” with long-term metabolic and neuroimmune maintenance therapies. By diversifying our molecular targets and mandating equitable, biomarker-driven clinical trial designs, the medical community can finally transform Alzheimer’s from a terminal decline into a manageable, chronic condition.

### 9.2. Key Takeaways for Research and Practice

For both research and clinical practice, the immediate path forward demands a definitive departure from single-target paradigms.

The primary takeaway for investigators is the urgent need to diversify the therapeutic pipeline. Future investigations must evaluate sequential combination therapies—pairing amyloid clearance with agents that target systemic neuroinflammation, endolysosomal repair, and metabolic dysfunction. Furthermore, clinical trials must enforce strict biomarker-driven enrollment while aggressively prioritizing historically underrepresented populations. A critical tension remains here: balancing the need for rigorous, biologically homogenous trial cohorts with the ethical mandate to test drugs on the diverse, multi-morbid populations who actually represent the real-world AD demographic.

In the clinic, the landscape has permanently shifted toward proactive precision medicine. Clinicians and neuroradiologists must tightly integrate advanced fluid biomarkers with high-resolution MRI protocols not only to confirm molecular pathology early but to rigorously mitigate life-threatening complications like ARIA. However, this proactive shift triggers a massive health-economics controversy: global healthcare systems are currently entirely unequipped—financially, logistically, and in terms of a specialized workforce—to support the serial neuroimaging, specialist infusions, and intense monitoring required to safely administer these therapies to millions of aging patients.

Ultimately, conquering Alzheimer’s disease requires intervening during the preclinical window and managing it continuously as a complex, multi-systemic condition, successfully shifting the paradigm from symptom palliation to lifelong biological preservation.

## Figures and Tables

**Table 3:** Differential diagnosis of alzheimer’s disease clinical phenotypes.

Phenotype	Clinical Hallmark	MRI Findings (Structural)	Metabolic/Molecular Imaging (PET)	Peripheral/Biofluid Markers	References
1. Amnestic (Classic)	Episodic memory impairment; progressive disorientation.	Medial Temporal Atrophy (MTA scale 3-4); hippocampal volume loss.	FDG-PET: Posterior cingulate and temporoparietal hypometabolism.	High p-tau217/p-tau181; Low Aβ42/ Aβ40 ratio.	[86,118]
2. Non-Amnestic (Atypical)	Visual (PCA), language (lvPPA), or executive deficits.	Focal atrophy (Parieto-occipital in PCA; Left temporal in lvPPA).	Tau-PET: High uptake in specific cortical hubs (outside hippocampus).	CSF biomarkers positive for AD pathology (A+/T+).	[93,96,119]
3. Neuropathic (Peripheral Focus)	Distal sensory loss; gait instability; autonomic dysfunction.	Often mild/non-specific global atrophy in early stages.	FDG-PET: Early signs of cerebral insulin resistance/global hypometabolism.	Reduced IENFD (Skin biopsy); axonal damage markers.	[120,121,122]
4. Mixed (CNS–PNS Crosstalk)	Global cognitive decline exacerbated by sensory-motor deficits.	Combined hippocampal and diffuse cortical thinning.	Integrated mismatch: Cortical hypometabolism + autonomic dysfunction markers.	Extremely high Plasma NfL; abnormal NCS/EMG studies.	[106,123]

**Table 4:** Summary of pivotal Phase 3 trials in anti-amyloid therapy.

Drug	Clinical Trial	Primary Outcome (Cognitive Decline)	Key Biomarker Insight	Safety Concern (ARIA)
Lecanemab	Clarity AD [19].	27% reduction at 18 months.	Amyloid-PET validated as a pharmacodynamic marker of plaque clearance.	12.6% ARIA-E; mostly asymptomatic.
Donanemab	TRAILBLAZER-ALZ 2 [20].	35% reduction (iADRS) in low/medium tau cohorts.	Start–stop dosing: treatment successfully ceased once PET confirmed amyloid negativity.	24% ARIA-E; markedly higher incidence in *APOE4* carriers.
Aducanumab	EMERGE/ENGAGE [154].	Significant in EMERGE (high-dose only); failed in ENGAGE.	Historic proof-of-concept for accelerated approval based on a surrogate endpoint.	41% ARIA-E in high-dose groups. (Commercially Discontinued)

## Data Availability

No new data were created or analyzed in this study. Data sharing is not applicable to this review article.

## Abbreviations

The following abbreviations are used in this manuscript:

Aβ	Amyloid-β
ABCA-1	ATP-binding cassette subfamily A member 1
AD	Alzheimer’s disease
ADLs	Activities of Daily Living
APP	Amyloid precursor protein
AQP-4	Aquaporin-4
ALPS	Analysis along the Perivascular Space
ALS	Amyotrophic Lateral Sclerosis
ARIA	Amyloid-Related Imaging Abnormalities
ARIA-E	Edema-ARIA
ARIA-H	Haemosiderin deposition-ARIA
ASOs	Antisense oligonucleotides
ATN	Amyloid-β/Tau/Neurodegeneration classification system
APOE	Apolipoprotein E gene
BBB	Blood–brain barrier
BNB	Blood–nerve barrier
BDNF	Brain-Derived Neurotrophic Factor
BPSD	Behavioral and psychological symptoms of dementia
CDK5	Cyclin-dependent kinase 5
CRISPR	Clustered regularly interspaced short palindromic repeats
CSF	Cerebrospinal fluid
CMAP	Attenuated compound muscle action potential
CNS	Central nervous systems
CRP	C-reactive protein
DMT	Disease-modifying therapy
DTI	Diffusion Tensor Imaging
EMA	European Medicines Agency
EMG	Electromyography
EOAD	Early-Onset Alzheimer’s Disease
FDA	Food and Drug Administration
FDG	Fluorodeoxyglucose
FLAIR	Fluid-attenuated inversion recovery
FTD	Frontotemporal Dementia
GFAP	Glial fibrillary acidic protein
GLP-1	Glucagon-like peptide-1
GRE	Gradient-recalled echo
gRNA	Guide RNA
GSK-3β	Glycogen synthase kinase-3β
HDR	Homology-Directed Repair
HOMA-IR	Homeostatic Model Assessment of Insulin Resistance
iADRS	integrated Alzheimer’s Disease Rating Scale
IENFD	Intraepidermal Nerve Fiber Density
ISF	Interstitial fluid
IL	Interleukin
HRQoL	Health-related quality of life
LATE	Limbic-predominant age-related TDP-43 encephalopathy
LOAD	Late-Onset Alzheimer’s Disease
LPS	Lipopolysaccharides
LTP	Long-Term Potentiation
lvPPA	Logopenic variant Primary Progressive Aphasia
MAPT	Microtubule-associated protein tau
MCID	Minimal Clinically Important Difference
MGBA	Microbiota–Gut–Brain Axis
MMPs	Metalloproteinases
MRI	Magnetic Resonance Imaging
mRNA	Messenger ribonucleic acid
MTA	Medial Temporal Atrophy
NCS	Nerve conduction studies
NHEJ	Non-Homologous End Joining
NIA-AA	National Institute on Aging–Alzheimer’s Association
NfL	Neurofilament Light Chain
NGF	Nerve Growth Factor
NGS	Next-Generation Sequencing
NLRP3	NOD-like receptor family pyrin domain-containing 3
OMIM	Online Mendelian Inheritance in Man
PCA	Posterior Cortical Atrophy
PET	Positron Emission Tomography
PNS	Peripheral nervous systems
P-TAU	Phosphorylated tau
ROS	Reactive oxygen species
SASP	Senescence-Associated Secretory Phenotype
SCFA	Short-chain fatty acid
SNAP	Sensory nerve action potential
sPDGFRβ	Soluble Platelet-Derived Growth Factor Receptor Beta
sTREM2	Soluble TREM2
SUVr	Standardized Uptake Value Ratio
TFEB	Transcription Factor EB
TNF-α	Tumor necrosis factor-α TNF-α
TREM2	Triggering receptor expressed on myeloid cells 2
WES	Whole Exome Sequencing
WGS	Whole Genome Sequencing
